# L5–S1 Anatomic Features Relevant to Minimally Invasive Decompression and Fusion: A Cadaveric and Imaging-Based Study

**DOI:** 10.3390/diagnostics16040610

**Published:** 2026-02-19

**Authors:** Miguel Relvas-Silva, André Rodrigues Pinho, Vitorino Veludo, Daniel Medina-Dias, António Pereira Rodrigues, Hélio Alves, Maria Dulce Madeira, Pedro Alberto Pereira

**Affiliations:** 1 Department of Orthopaedics and Traumatology, ULS São João, Alameda Professor Hernâni Monteiro, 4200-319 Porto, Portugal; arpcinco@hotmail.com (A.R.P.); vmveludo@gmail.com (V.V.); danielmedinadias@gmail.com (D.M.-D.); 2 Department of Surgery and Physiology, Faculty of Medicine, University of Porto, Alameda Professor Hernâni Monteiro, 4200-319 Porto, Portugal; 3 NeuroGen Research Group, Center for Health Technology and Services Research (CINTESIS), Rua Dr. Plácido da Costa, 4200-450 Porto, Portugal; madeira@med.up.pt (M.D.M.); pedroper@med.up.pt (P.A.P.); 4 Unit of Anatomy, Department of Biomedicine, Faculty of Medicine, University of Porto, Alameda Professor Hernâni Monteiro, 4200-319 Porto, Portugal; antoniojpr10@gmail.com (A.P.R.); helioalves@med.up.pt (H.A.); 5 CINTESIS@RISE (Center for Health Technology and Services Research@Health Research Network), Faculty of Medicine, University of Porto, Alameda Professor Hernâni Monteiro, 4200-319 Porto, Portugal

**Keywords:** L5–S1 segment, lumbosacral anatomy, extraforaminal, transforaminal, minimally invasive spine surgery, endoscopic surgery, iliolumbar ligament

## Abstract

**Background/Objectives**: The L5–S1 segment presents unique characteristics that make surgical access challenging in minimally invasive spine surgery (MISS) procedures. Variability in bony and neural anatomy may restrict transforaminal and extraforaminal approaches, yet few studies have combined cadaveric dissection with radiologic analysis to define relevant morphology in L5–S1 approaches. The purpose of the study is to characterize anatomical and radiological features of the lumbosacral region relevant to MISS planning and execution. **Methods**: Twelve Thiel-embalmed donor bodies underwent CT imaging (lumbopelvic region) followed by posterior dissection. Bony landmarks were used to obtain bilateral anatomical measurements. Qualitative anatomical analysis included iliolumbar ligament morphology and extraforaminal access feasibility. CT-based morphometrics included L5 transverse process (TP) length; maximal and minimal distances between L5 TP and sacral ala; extraforaminal area bounded by L5 TP, L5–S1 facet (zygapophyseal) joint, and sacral ala; iliac crest-based approach angle to the L5–S1 intervertebral disc (IVD); minimal distance between this approach vector and the ventral ramus of the fifth lumbar spinal nerve (VRL5); facet angulation; and iliac crest height. **Results**: No left–right asymmetry was detected. Except for L5 TP length, all anatomical measurements obtained directly in the donor bodies differed significantly between sexes. A direct IVD access with a uniportal endoscopic working tube was feasible in 25% of cases. On CT analysis, the maximal and minimal distances between the L5 TP and sacral ala were 11.1 (4.0) mm and 5.6 ± 2.9 mm, with a mean extraforaminal area of 202.0 ± 45.9 mm^2^. The mean approach angle was 35.2 ± 5.0°, and an extraforaminal corridor to L5–S1 IVD was feasible in 75% of donated bodies. The median minimal distance between the approach vector and the VRL5 was 5.0 (7.1) mm, with frequent overlap. **Conclusions**: The results of this study reveal that the L5–S1 segment shows substantial interindividual morphologic variability, compromising the feasibility of transforaminal and extraforaminal MISS approaches, and highlight the need for individualized preoperative planning, neural identification and/or bony resection to create a safe working corridor.

## 1. Introduction

Commonly occurring degenerative spinal conditions such as intervertebral disc (IVD) herniation, disc degeneration and/or spondylolisthesis occur most frequently in the lower lumbar spine, particularly at the L4–L5 and L5–S1 levels [1,2]. The L5–S1 segment has distinct anatomical and biomechanical characteristics that may contribute to these degenerative diseases [2]. Positioned at the transition between the mobile lumbar spine and the rigid sacrum, this segment is subject to substantial compressive and shear forces [3,4]. It typically exhibits the greatest IVD height, more coronally oriented facet (zygapophyseal) joints, and stabilizing structures such as the iliolumbar ligament, contributing to its unique functional profile [1,2,3,5,6,7].

Surgical management of lumbar degenerative disease and spinal instability involves decompression and/or fusion [8,9]. When surgery is required, the aforementioned features may influence the choice of surgical technique and approach [9,10,11]. For the L5–S1 level, posterior approaches remain widely used due to surgeon familiarity and versatility [12]. The ongoing demand to maximize clinical outcomes while minimizing morbidity has driven the rapid expansion of minimally invasive spine surgery (MISS), including tubular and endoscopic techniques, as they may reduce iatrogenic muscle injury, blood loss and postoperative pain, promoting faster recovery [13,14,15,16,17,18,19].

The L5–S1 IVD space access may be performed using interlaminar, transforaminal (TF) and extraforaminal (EF) approaches. A relatively wide interlaminar window at L5–S1 facilitates posterior access and may have direct clinical implications for surgical approach selection and outcomes in lumbosacral procedures [20,21]. In contrast, L5–S1 TF and EF access, typically used in decompression and fusion procedures for the management of foraminal and EF IVD herniations, far-out syndrome, lumbar stenosis and/or spondylolisthesis, can be particularly challenging due to complex regional anatomy and morphologic variability [15,22,23,24,25,26]. For instance, the iliac crest height, foraminal dimensions, and orientation of bony structures may restrict working angles and limit safe instrument trajectories [22,27].

To the best of our knowledge, few studies have specifically analyzed the L5–S1 segment morphologic features in the surgical setting [22,28,29]. This study aims to characterize anatomical and radiological features of the above-mentioned segment and to identify factors relevant for the planning and execution of MISS procedures.

## 2. Materials and Methods

This study was conducted at the Unit of Anatomy, Department of Biomedicine, Faculty of Medicine of the University of Porto (FMUP), Portugal. Ethical approval was granted by the Ethics Committee of FMUP/RISE Health on 23 September 2025 (365/CEFMUP-RISE Health/2025).

Twelve donor bodies preserved using Thiel’s embalming method [30] were included in the present study. All donor bodies were derived from donation with informed consent, written and signed by the donors themselves, in accordance with Portuguese Decree-Law n° 271/99. Age and gender were retrieved from donation records. Donor bodies were excluded if they presented unexplained deformities or signs of previous dissection/surgery involving the lumbosacral region.

Prior to anatomical dissection, computed tomography (CT) scans of the lumbopelvic region were obtained for all donor bodies. All predefined measurements were obtained bilaterally (totalizing 24 segments) and were independently recorded by two observers.

### 2.1. Anatomical Dissection

**Figure 1:**
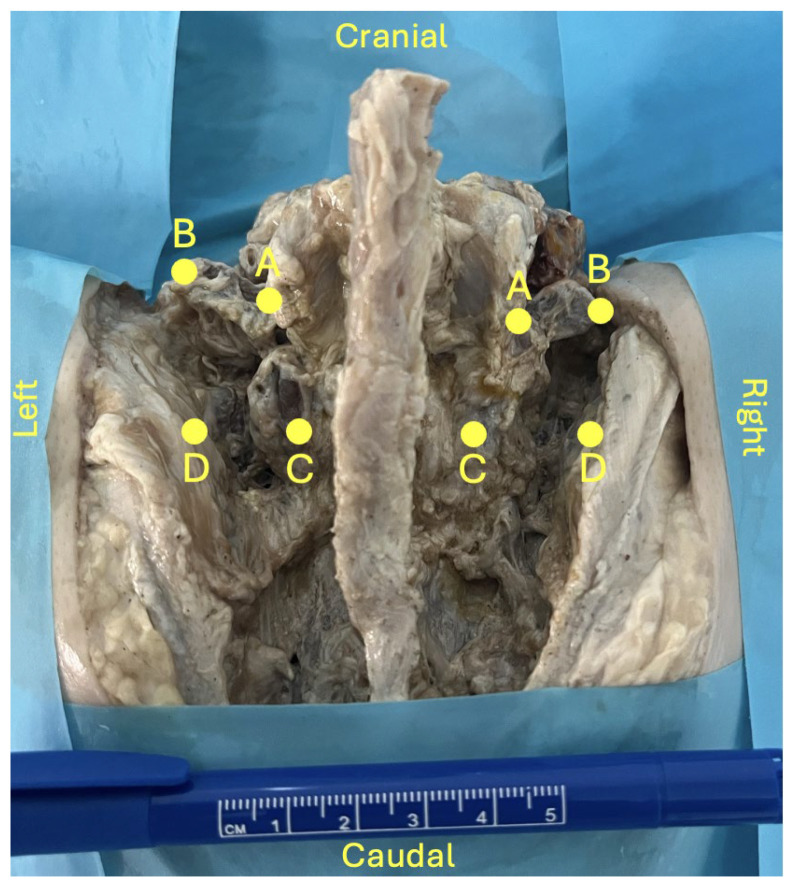
Lumbosacral region dissection (posterior view). For demonstration purposes, the L5–S1 zygapophyseal joint was partially opened on the left side. A: base of the L5 transverse process (TP); B: tip of the L5 TP; C: inferior tip of the L5 inferior articular process; D: medial margin of the iliac crest at the level of point C.

A standard midline posterior approach to the lumbosacral spine was performed to expose the posterior elements from L4 to S2 vertebrae. Care was taken to preserve bony structures as well as the native position of ligamentous and neurovascular components. Four predefined anatomical landmarks (points A–D) were identified (Figure 1):

- Point A: base of the L5 transverse process (TP);
- Point B: tip of the L5 TP;
- Point C: inferior tip of the L5 inferior articular process;
- Point D: medial margin of the iliac crest at the level of point C.

Using these landmarks, four distances were measured (A–B, C–D, A–C and B–D) using a ruler graded in millimetres.

**Figure 2:**
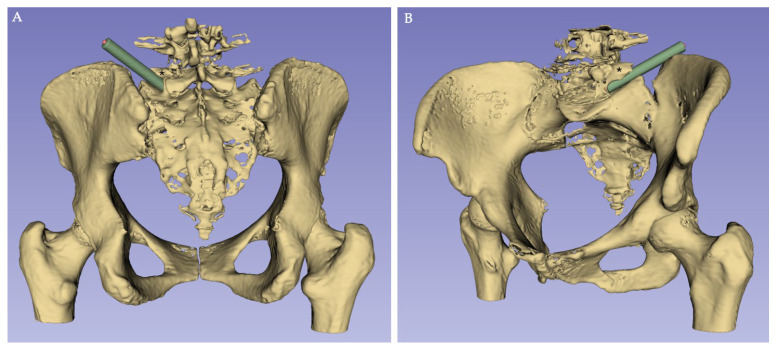
3D reconstruction of CT scan images (using 3D Slicer Software, version 5.10.0), with a representation of an endoscopic L5–S1 disc access ((**A**), posterior view; (**B**), anterolateral view); *: L5 transverse process; green cylinder: schematic representation of the endoscopic working tube.

Qualitative assessments included the characterization of the iliolumbar ligament anatomy from a posterior view, regarding its attachment points, number of bands and shape. Moreover, direct access to the L5–S1 IVD through TF and/or EF approaches using a uniportal endoscopic working tube (Joimax^®^ GmbH, Karlsruhe, Germany, Tessys, REF WTS176575; 170 mm length; 6.5 mm inner/7.5 mm outer diameter) was tested (Figure 2) to assess potential anatomical barriers.

### 2.2. Imaging Analysis

**Figure 3:**
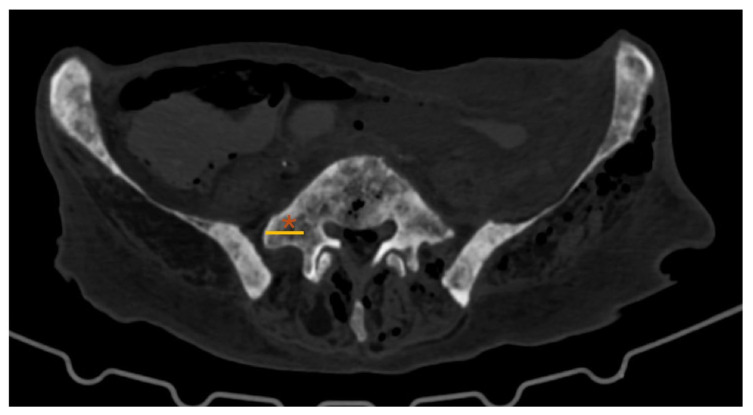
Lumbar spine CT—axial plane; *: L5 transverse process (TP); yellow line: L5 TP length.

**Figure 4:**
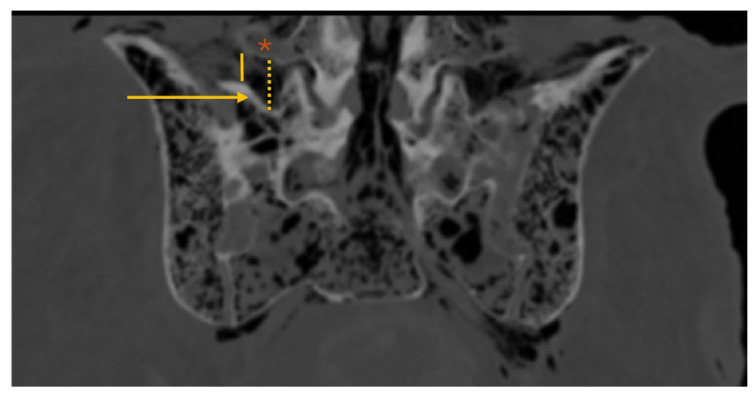
Lumbosacral spine CT—coronal plane; *: L5 transverse process (TP); arrow: sacral ala; dotted yellow line: maxL5TP–sacrum (maximum distance from the inferior border of the L5 TP to the superior border of the sacral ala); solid yellow line: minL5TP–sacrum (minimum distance from the inferior border of the L5 TP to the superior border of the sacral ala).

**Figure 5:**
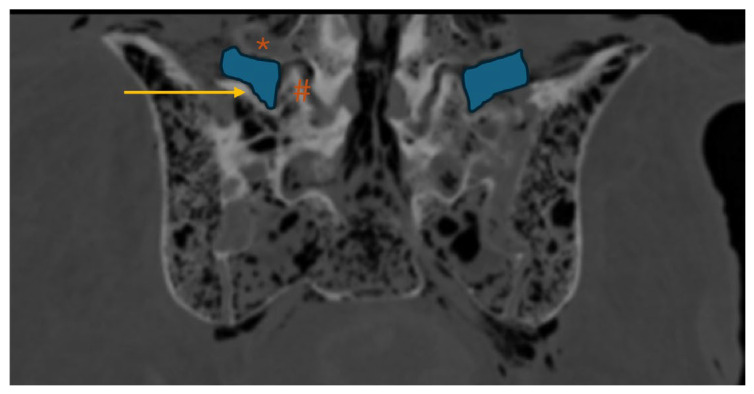
Lumbosacral spine CT—coronal plane; *: L5 transverse process (TP); #: L5–S1 facet joint; arrow: sacral ala; blue area: EF (extraforaminal) area (EF area bounded superiorly by the L5 TP, medially by the L5–S1 facet joint and inferiorly by the sacral ala).

**Figure 6:**
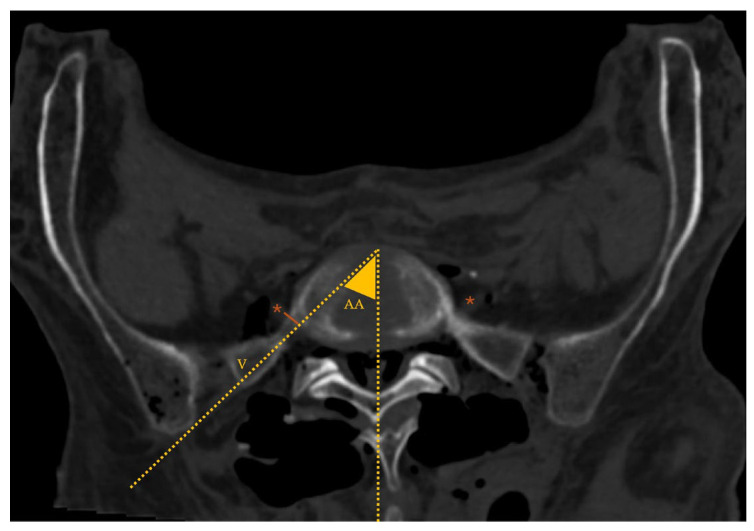
Lumbosacral spine CT—axial plane; *: ventral ramus of the fifth lumbar spinal nerve (VRL5); dotted yellow lines: marking of the approach angle (AA; angle between the midline and the vector (V) tangential to the iliac crest that crosses the most anterior point of the L5–S1 IVD space; solid orange line: minVRL5–V (minimum distance from the VRL5 to vector line).

Using Sectra^®^ IDS7 Software (version 24.2.22.6193), five morphometric parameters were defined and measured on CT scan images (Figure 3, Figure 4, Figure 5 and Figure 6):

- L5–TP: length of the L5 TP (axial plane; Figure 3);
- MaxL5TP–sacrum and minL5TP–sacrum: maximum and minimum distance from the inferior border of the L5 TP to the superior border of the sacral ala, respectively, (coronal plane intersecting the L5 TP centre; Figure 4);
- EF area: EF area bounded superiorly by the L5 TP, medially by the L5–S1 facet joint and inferiorly by the sacral ala (coronal plane; Figure 5);
- Approach angle (AA): angle between the midline and the vector line (V) tangential to the iliac crest that crosses the most anterior point of the L5–S1 IVD space (axial plane at L5–S1 IVD space; Figure 6);
- MinVRL5–V: minimum distance from the ventral ramus of the fifth lumbar spinal nerve (VRL5) to V (axial plane; Figure 6).

**Figure 7:**
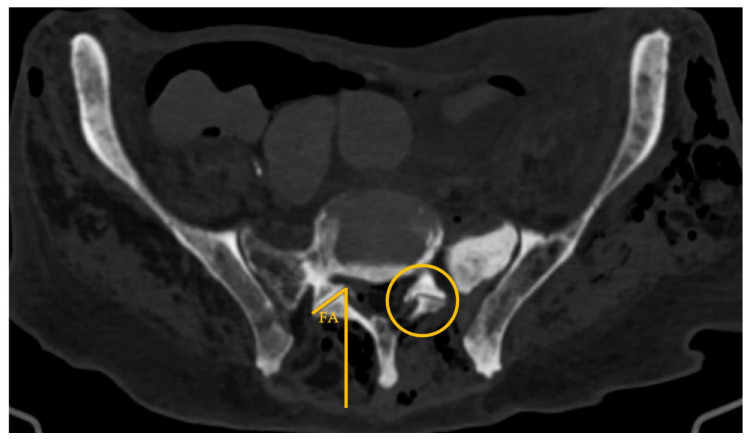
Lumbosacral spine CT—axial plane; circle: highlights the location of the left L5–S1 facet (zygapophyseal) joint; FA: facet joint angulation, as defined by the yellow solid lines.

Further analysis included evaluation of facet joint angulation, measured as the angle between a sagittal line and a line crossing the L5–S1 zygapophyseal joint (Figure 7), assessment of iliac crest height (using coronal CT cuts and considering the classification described by Nagasse and colleagues [31]), and a qualitative assessment of sacral morphology (based on the characteristics described by Miller and Routt [32]).

### 2.3. Statistical Analysis

Statistical analyses were performed using IBM SPSS^®^ Software, version 27. Results for continuous variables are expressed as medians (IQR [interquartile range]) or means (±SD [standard deviation]) for non-normal or normal variable distribution, respectively. Categorical variables were expressed as absolute and relative frequencies. Paired Student’s *t*-tests, Mann–Whitney U test and Spearman’s correlation coefficients were applied to analyze continuous variables. Interobserver reliability was assessed using intraclass correlation coefficients (ICCs) and their 95% confidence intervals. ICC estimates were based on a mean rating (k = 2), absolute agreement, and a two-way mixed-effects model [33,34]. *p*-values less than 0.05 were considered statistically significant.

## 3. Results

Twelve donor bodies were included (eight male, four female), all Caucasian and of Portuguese nationality, with a mean age at death of 75 ± 9 years. Inter-rater reliability ranged from 0.816 to 0.995. No statistically significant left–right asymmetry was evident between measurements.

### 3.1. Anatomical Dissection Results

No statistically significant sex-related differences were observed for L5–TP (A–B distance). In contrast, all other measurements showed statistically significant differences between female and male donor bodies:

- A–B distance: 16 ± 6 mm (female) vs. 15 ± 2 mm (male); *p* = 0.702;
- C–D distance: 16 ± 5 mm (female) vs. 10 ± 3 mm (male); *p* = 0.003;
- A–C distance: 18 ± 4 mm (female) vs. 27 ± 6 mm (male); *p* < 0.001;
- B–D distance: 21 ± 6 mm (female) vs. 29 ± 7 mm (male); *p* = 0.01.

From a posterior view, the iliolumbar ligament originated from L5 TP bilaterally in all donor bodies. Morphologically, it consisted either of a single (n = 18) or a double band (n = 6), displaying a rectangular (n = 16) or fan-shaped (n = 8) configuration.

Direct access to the L5–S1 IVD using an endoscopic approach was feasible in 25% of attempts (n = 6/24), with no gender difference (*p* = 0.545).

### 3.2. Imaging Results

Summary data is presented in Table 1 and Table 2.

Imaging analysis showed an equal distribution of nondysmorphic (n = 6) and dysmorphic (n = 6) sacra.

CT-based L5–TP (Figure 3) closely matched the anatomical A–B distance, with no significant differences (*p* = 0.144). However, the values obtained in the CT analysis were slightly higher than those obtained directly in donor bodies.

The maxL5TP–sacrum distance (Figure 4) was 11.1 (4.0) mm, consistently located at the level of the second quarter of the L5 TP (from its base). The mean minL5TP–sacrum distance (Figure 4) was 5.6 ± 2.9 mm, always near the L5 TP tip. No statistically significant differences were observed between nondysmorphic and dysmorphic sacral morphology (*p* = 0.662 and *p* = 0.498, respectively).

The mean CT-defined EF area (Figure 5) was 202.0 ± 45.9 mm^2^. No statistically significant difference was found between nondysmorphic and dysmorphic sacral morphologies (*p* = 0.540): 193.8 ± 53.2 and 212.0 ± 38.7 mm^2^, respectively.

The mean AA (Figure 6) was 35.2 ± 5.0°. An EF vector (V) tangential to the iliac crest that crosses the most anterior point of the L5–S1 IVD space defined a workable corridor to the L5–S1 IVD in 75% of the cases (18/24 sides). These cases had significantly greater AA (29.9 ± 3° vs. 37.2 ± 4°; *p* = 0.02) and, despite not being statistically significant, larger L5 TP-to-sacrum distances, greater EF areas, and increased minVRL5–V values were found. However, in 44% (8/18) of cases with a workable corridor on CT, the distance from V to the VRL5 was less than 5 mm.

The median minVRL5–V distance was 5.0 (7.1) mm, with no statistically significant gender (*p* = 0.788) or sacral morphology (*p* = 0.429) variation. This parameter was zero in 8 out of 24 cases (33%), meaning direct overlap between the V and the VRL5.

The mean L5–S1 facet joints angulation (Figure 7) was 51.4 ± 10.8°, with no statistically significant difference between cases in which EF access was feasible and those in which it was not (*p* = 0.597). Iliac crest height was graded 3 to 7, with 58% of cases (7/12) grading 6 or 7. Further analysis evaluated the correlation between iliac crest height and AA—Spearman’s ρ = −0.465, *p* = 0.150.

## 4. Discussion

This study provides an integrated anatomical and radiological characterization of the lumbosacral junction relevant to posterior TF and EF access using MISS techniques. Although prior studies have highlighted the challenges of L5–S1 segment access due to, for instance, iliac crest, sacral and facet morphology [22,27,35,36], to the best of our knowledge, few have combined cadaveric dissection and imaging analysis [37,38].

Regarding dissection data, there was considerable gender-related variation, with no side-to-side asymmetry. The C–D distance, which reflects the spatial relationship between the inferior tip of the L5 inferior articular process and the medial margin of the iliac crest, was greater in females, whereas the A–C and B–D vertical distances (from the base of the L5 TP to the inferior tip of the L5 inferior articular process and from the L5 TP tip to the medial margin of the iliac crest, respectively) were greater in males. These differences may reflect sexual dimorphism in stature and pelvic morphology [3,39,40,41]. From a surgical standpoint, such variations may influence instrument trajectory and available working space.

Qualitative assessment of iliolumbar ligament and endoscopic access provides useful information. Regarding the iliolumbar ligament anatomy, our findings closely align with results from Dabrowski and Ciszek [42], and may help surgeons in preventing its iatrogenic injury, which may compromise lumbosacral stability. The relatively low direct accessibility (25%) to the L5–S1 IVD using a uniportal endoscopic working tube further underscores the need for detailed preoperative planning. The angulation of the IVD space, combined with variable bony morphology, may condition this access and request bony gestures—such as L5 TP resection, sacral ala resection and/or foraminoplasty—or alternative approaches to access the IVD space. To overcome some of the limitations imposed by the unique anatomy of the L5–S1 segment, a transiliac approach has been described [37,38,43]. Notably, in a cadaveric study, Sousa and colleagues demonstrated that L5–S1 transiliac intraforaminal lumbar interbody fusion is a feasible surgical technique, which allows both a more centrally placed interbody cage in the coronal plane without compromising the anterior position in the lateral plane, and also the preservation of the integrity of the major anatomic structures at risk [38]. In this context, Sousa and collaborators recently showed the outcomes of five patients submitted to a transiliac endoscopic-assisted L5–S1 intraforaminal lumbar interbody fusion with good clinical results, as well as high fusion rates at 12 months [44]. However, they stated that this surgical technique may present some complications, such as late-onset dysesthesia of the ipsilateral lower limb (10 to 14 days after surgery) [44].

The CT-based component of the study adds further nuance to the anatomical data analysis. Neither gender nor sacral morphology statistically significantly influenced the measurements. Nonetheless, male donor bodies presented greater values for maxL5TP–sacrum, minL5TP–sacrum, EF area and minVRL5–V, while females had greater AA, which may reflect sexual dimorphism in stature and pelvic morphology, as previously suggested by the results obtained directly from anatomical dissection analysis.

The EF area, defined using bony and articular landmarks easily identifiable during surgical procedures, is relevant for EF access and decompression procedures, and clinically relevant in scenarios such as far-out syndrome [23,24]. This area exhibited wide variability (123–287 mm^2^), which may reflect interindividual anatomical variations and/or the presence of degenerative changes.

The mean AA value and L5–S1 facet joints angulation are comparable to values from other anatomical series [22]. According to CT-based planning, the EF corridor to the L5–S1 IVD was feasible in 75% of donor bodies segments. This value widely differs from the cadaveric findings for a direct endoscopic access (feasible in only 25% of cases), which may highlight the need for multiplanar CT analysis to understand the local morphology. Potential barriers to the L5–S1 segment EF approach might include iliac crest and facet joint morphology, and L5 TP to sacrum distances. The moderate negative correlation between iliac crest height and AA reinforces the iliac crest as a frequent limiting factor when planning EF trajectories, similar to previous works [35,36]. Contrarily, facet joint orientation showed no statistically significant differences between donor segments in which EF access was feasible and those in which it was not. Authors believe articular processes degeneration, with osteophytosis and hypertrophy, may impede access more than facet angulation. Therefore, partial resection of the isthmus, partial facetectomy and/or foraminoplasty may improve access during EF approaches.

The minVRL5–vector distance was generally short, with median values around 5.0 (7.1) mm. In 33% of the cases, the projected vector directly collided with the VRL5. This finding reinforces the need for meticulous neural identification and protection. Considering that most interbody cages measure close to 10 mm in width, even minor deviations between planned and real trajectories may place the VRL5 at risk.

Collectively, this work provides quantitative insight into the geometric constraints of the L5–S1 surgical corridor for TF and EF approaches, which may be useful for the management of foraminal and EF IVD herniations, foraminal stenosis and far-out syndrome, revision cases (to avoid scar tissue) and/or lumbar interbody fusion procedures. Considerable interindividual variability in parameters that define the L5–S1 surgical working corridor, together with the close proximity of the VRL5, reinforce the need for individualized preoperative analysis, customized approaches and real-time neuromonitoring (when applicable). Combining the values of maxL5TP–sacrum, minL5TP–sacrum and AA, and the dimensions of commonly used MISS (including endoscopic) working tubes, these findings suggest that bony gestures, or even the choice for alternative approaches, will frequently be necessary to optimize procedures and minimize iatrogeny.

We acknowledge some limitations and strengths to the present study. As with all cadaveric studies, tissues may not accurately replicate the physiological and biomechanical properties of living specimens, as death and/or embalming procedures can alter tissue consistency, potentially affecting dissection and measurement accuracy (as suggested by slightly but non-significant differences in L5 TP length measurements between cadaveric and imaging analysis; *p* = 0.144). However, our measurements were based in fixed bony and articular landmarks which may barely be affected by death and/or preservation methods. Moreover, examiners were trained in the local anatomy and independent measurements were performed, with a good-to-excellent inter-rater reliability. Furthermore, we measured some parameters previously reported in the literature, and the results obtained were similar to this data, which unequivocally provides robustness to our results. Another relevant limitation concerns the small sample of Portuguese Caucasian and older donor bodies, limiting generalizability and statistical power to detect subtle differences. Finally, the absence of accurate donor height and weight prevented reliable correlation of morphological variability with these anthropometric parameters. A major strength of this study is the combined use of CT imaging and direct anatomical measurements, allowing validation of imaging-based assumptions with high-fidelity Thiel-preserved tissue. Bilateral measurements and independent observers further reinforced reliability, a fact corroborated by ICC values in the good–excellent range. 

Future investigations should involve larger and more heterogeneous cohorts to improve generalizability and statistical power. The integration of three-dimensional reconstructions with navigation-assisted planning may allow more accurate visualization of individual L5–S1 access corridors and facilitate the definition of threshold anatomical values for variables identified in this study. Moreover, advances in instrument and implant design, including the development of L5–S1-specific endoscopic working tubes and smaller-profile or expandable interbody devices, may expand surgical options for anatomically challenging cases. Correlating patient-specific anatomical features with surgical feasibility, complication profiles, and clinical outcomes—using patient reported outcome measures—for both decompression and fusion procedures would further refine patient selection and procedural planning. Given growing concerns regarding radiation exposure during fluoroscopy-guided procedures, future studies also evaluating alternative methods to identify and establish access to the working corridor to the L5–S1 segment may be performed. In this context, for instance, Zhang and colleagues demonstrated that ultrasound-guided TF percutaneous endoscopic lumbar discectomy (L3–L4, L4–L5 and L5–S1 levels) is a valuable method that largely reduces radiation doses while providing treatment effects comparable to those achieved with conventional fluoroscopic guidance [45]. Indeed, this is a field of research with great relevance for both patients and healthcare professionals, and thus, further comparative studies could be performed combining both techniques.

## 5. Conclusions

In conclusion, the lumbosacral junction exhibits considerable variability in bony morphology and specific neural relations, yet consistent anatomical relationships can be defined using reproducible landmarks. The integration of imaging and cadaveric findings, and the recognition of segment specificities, enhances understanding of the L5–S1 surgical corridor. Preoperative assessment of the distance between the inferior border of the L5 transverse process and the superior border of the sacral ala, the approach angle to a transforaminal or extraforaminal access to L5–S1 intervertebral disc space and/or the precise location of ventral ramus of the fifth lumbar spinal nerve may assist surgeons in planning safer, more effective, minimally invasive access routes.

## Figures and Tables

**Table 1:** Analysis of CT scan parameters (global results and by gender).

	Total	Female	Male	*p*-Value Female vs. Male
L5–TP (mm; mean ± SD)	17.8 ± 3.1	19.4 ± 3.1	16.8 ± 2.8	0.197
MaxL5TP–sacrum (mm; median (IQR))	11.1 (4.0)	9.9 (3.9)	11.4 (4.6)	0.315
MinL5TP–sacrum (mm; mean ± SD)	5.6 ± 2.9	4.8 ± 1.2	6.0 ± 3.6	0.498
EF area (mm^2^; mean ± SD)	202.0 + 45.9	180.0 ± 9.8	214.8 ± 54.2	0.540
AA (°; mean ± SD)	35.2 ± 5.0	38.8 ± 4.8	33.2 ± 4.0	0.563
MinVRL5–V (mm; median (IQR))	5.0 (7.1)	2.7 (9.1)	5.0 (7.2)	0.788

Detailed variables characterization is available in Section 2. AA—approach angle; EF—extraforaminal; IQR—interquartile range; L5–TP—length of the L5 TP; Max—maximum; Min—minimum; SD—standard deviation; TP—transverse process; V—vector; VRL5—ventral ramus of the fifth lumbar spinal nerve.

**Table 2:** Analysis of CT scan parameters regarding sacral morphology.

	Nondysmorphic	Dysmorphic	*p*-Value
L5–TP (mm; mean ± SD)	18.5 ± 4.1	16.9 ± 0.56	0.392
MaxL5TP–sacrum (mm; median (IQR))	11.0 (4.6)	11.1 (5.5)	0.662
MinL5TP–sacrum (mm; mean ± SD)	5.0 ± 3.1	6.2 ± 2.8	0.498
EF area (mm^2^; mean ± SD)	193.8 ± 53.2	212.0 ± 38.7	0.540
AA (°; mean ± SD)	34.4 ± 4.5	36.2 ± 5.8	0.563
MinVRL5–V (mm; median (IQR))	6.0 (10.9)	2.6 (6.1)	0.429

Detailed variables characterization is available in Section 2. AA—approach angle; EF—extraforaminal; IQR—interquartile range; L5–TP—length of the L5 TP; Max—maximum; Min—minimum; SD—standard deviation; TP—transverse process; V—vector; VRL5—ventral ramus of the fifth lumbar spinal nerve.

## Data Availability

The raw data supporting the conclusions of this article will be made available by the authors on request.

## Abbreviations

The following abbreviations are used in this manuscript:

AA	Approach angle
CT	Computer tomography
EF	Extraforaminal
EF area	EF area bounded superiorly by the L5 TP, medially by the L5–S1 facet joint and inferiorly by the sacral ala
IVD	Intervertebral disc
L5	Fifth lumbar vertebra
MaxL5TP–sacrum	Maximum distance from the inferior border of the L5 TP to the superior border of the sacral ala
MinL5TP–sacrum	Minimum distance from the inferior border of the L5 TP to the superior border of the sacral ala
MinVRL5–V	Minimum distance from the VRL5 to V
MISS	Minimally invasive spine surgery
S1	First sacral vertebra
TF	Transforaminal
TP	Transverse process
V	Vector line tangential to the iliac crest that crosses the most anterior point of the L5–S1 IVD space
VRL5	Ventral ramus of the fifth lumbar spinal nerve
